# Journal of diabetes and metabolic disorders: launch editorial

**DOI:** 10.1186/2251-6581-11-1

**Published:** 2012-08-02

**Authors:** Bagher Larijani

**Affiliations:** 1 Endocrine and Metabolism Research Institute, Tehran University of Medical Sciences, Tehran, Iran

## 

The high prevalence and great burden of diabetes and metabolic disorders are part of a growing problem of chronic disease predominance in the contemporary society. The World Health Organization has identified diabetes to be globally “epidemic”, and points out the need to increase awareness of this status [1]. The chronic nature of these diseases, their long term impact on both patients and their families, and the fact that a definitive “cure” is still missing justifies the involvement of a large number of researchers and clinicians in these areas. This in turn results in numerous publications as a mean of spreading the gathered experiences and obtained knowledge and ultimately aiming to improve the patients’ well-being. The *Journal of Diabetes & Metabolic Disorders* (JDMD) is launched with the hope of contributing to the efforts made to reach this goal.

JDMD is the continuation of our previous publication, the Iranian Journal of Diabetes and Lipid Disorders, an electronic-only journal that published a total of 63 articles between 2008 and 2011. JDMD is owned by the Endocrinology and Metabolism Research Institute (EMRI), a center of excellence and a World Health Organization (WHO) collaborator in the prevention, diagnosis and management of Diabetes and Osteoporosis. EMRI is affiliated to Tehran University of Medical Sciences which is the first and most outstanding academic medical center at national level. The journal will cover a broad range of subjects related to endocrinology such as clinical and epidemiological studies on metabolic diseases and their complications, as well as novel and emerging therapeutics and related basic sciences studies, to name a few specific subjects. Two particular topics, namely “Aging” and “Interdisciplinary Research” are intended to be the subjects of special issues. Like its predecessor, JDMD is a peer-reviewed journal and, after acceptance, articles will be immediately published online, and soon after indexed in PubMed. All articles submitted to JDMD will be peer reviewed by our Editorial Board (http://www.jdmdonline.com/about/edboard) and, after acceptance, will be immediately published online, and soon after indexed in PubMed.

The aim of the changes made to the journal is to move to a more dynamic scheme of publication, which will be more in line with the current modes of scientific communication and spread of health-related information. With the wide dissemination of new communication technologies, and the trend toward “open science”, methods are debated to make the best from new models of data publishing [2]. As an open access journal, JDMD articles will be freely available to all potential readers, leading to a broader audience and higher visibility of articles [3]. Presently, although JDMD is an open-access journal, all publishing costs are covered by the Tehran University of Medical Sciences.

A multidisciplinary approach to endocrinology problems has been a central paradigm in the research activities of EMRI during the past seventeen years; the same spirit is intended to be present in the journal, which will propose a variety of themes. We rely on the distinguished scientists that form the Editorial Board to ensure the quality of published articles in these diverse subjects.

Finally, a journal’s success and impact depends directly on the publication of the hard work of researchers in the field. As such, we would welcome you to submit your research to *Journal of Diabetes & Metabolic Disorders* in order to promote it towards becoming one of the top journals in its field.
